# Symptoms and Outcomes in Colorectal Cancer Patients: A Saudi Tertiary Hospital's Five-Year Analysis

**DOI:** 10.7150/jca.116682

**Published:** 2025-08-11

**Authors:** Abdossalam M. Madkhali, Mohammed A. Almozini, Hasah F. Alaluan, Mohammed M. Ahmed, Mohammed H. Alnajeim, Nada F. Alsaif, Shatha K. Bin Dher, Ahmed M. Alsultan, Tareq Salah

**Affiliations:** 1 Department of Medicine, Oncology, Hematology and Radiation Oncology Unit, College of Medicine, King Saud University, Riyadh, Saudi Arabia.; 2 Primary Care, Riyadh Second Health Cluster, Riyadh, Saudi Arabia.; 3 College of Medicine, King Saud University, Riyadh, Saudi Arabia.; 4 Department of Oncology, Prince Sultan Medical City, Riyadh, Saudi Arabia.; 5 Clinical Oncology and Nuclear Medicine Department, Faculty of Medicine, Assiut University, Assiut, Egypt.

**Keywords:** oncology, colorectal cancer, radiation oncology, colon, rectal

## Abstract

**Background:** Colorectal cancer (CRC) is the third most common cancer worldwide. In Saudi Arabia, the 2020 cancer incidence report found that CRC was the most common cancer among men and had the highest mortality rate. Given the correlation between cancer symptom awareness and early detection and recognizing the significance of patient history in CRC diagnosis, this study aims to identify the presenting symptoms of CRC, assess survival by stage across the population, and better understand disease demographics in Saudi Arabia.

**Methods:** We conducted a retrospective cohort analysis of 655 patients with CRC diagnosed between 2016 and 2020, inclusive. The cancer registry database at King Khalid University Hospital was used to retrospectively collect data from electronic records. Various relevant data were extracted and analyzed.

**Results:** The results showed that the most common presenting symptom was abdominal pain (329, 50.2%), followed by weight loss (262, 40%), hematochezia (rectal bleeding or blood in stool) (242, 36.9%), and anemia (238, 36.3%). The overall three-year survival rate was 77.6%. For stages I, II, III, and IV it was 100%, 91.9%, 86.4%, and 61.8%, respectively. with a significant difference (p = 0.0001).

**Conclusions:** Rectal bleeding and other “alarming symptoms” were observed in fewer than 40% of the studied population. In the cohort, only one patient was diagnosed via a screening colonoscopy. Study also confirmed that survival improved with earlier stages at diagnosis. Encouraging preventative measures, raising awareness of CRC, and improving access to screening, could all contribute to earlier identification, reduced staging, and a better prognosis.

## Introduction

Colorectal cancer (CRC) is the third most common cause of cancer-related deaths globally, with an increasing incidence in developing countries 1, 2. In Saudi Arabia, CRC was the most frequently diagnosed and fatal cancer among men in 2020, the third most common cancer among females, and the second most common overall 2.

In a retrospective cohort study of over 29,000 patients referred by general practitioners to an outpatient colorectal surgery clinic over a 22-year period, the presenting symptoms among the 1,626 patients who were eventually diagnosed with colon cancer were as follows: A change in bowel movements was the most prevalent symptom (74%). The combination of rectal bleeding and changes in bowel habits was the most common symptom pairing, occurring in 51% of all malignancies and 71% of cases involving rectal hemorrhage. Other noted symptoms included rectal mass (24.5%), abdominal mass (12.5%), and iron-deficiency anemia **)**9.6%), while abdominal pain was the least prevalent symptom **)**3.8%) 3.

The five-year survival rate for patients with CRC is approximately 65% 4, and the stage of the disease at diagnosis is the most significant prognostic factor. Data from the Surveillance, Epidemiology, and End Results database for the period between 1991 and 2000 indicated the following five-year survival rates for colon cancer, stratified by stage: 93% for Stage I (T1-2N0), 85% for Stage IIA (T3N0), 72% for Stage IIB (T4N0), 83% for Stage IIIA (T1-2N1), 64% for Stage IIIB (T3-4N1), 44% for Stage IIIC (N2), and 8% for Stage IV 5. Another study reported a decline in five-year survival rates from 90% for individuals with early-stage cancer to just 14% for those with unresectable metastatic disease 6.

A matched case-control study of 5,075 incident cases of early-onset CRC among US commercial insurance policyholders (113 million persons aged 18-64) who were diagnosed between 2006 and 2015 and had been continuously enrolled for at least two years revealed a significant association between several symptoms—including abdominal discomfort, rectal bleeding, diarrhea, and iron-deficiency anemia—and an elevated risk of early-onset CRC 7. These symptoms began between three months and two years prior to diagnosis. This highlights the fact that symptoms may manifest well before the diagnosis date, potentially leading to more advanced disease at the time of identification and necessitating more aggressive therapy 6. Therefore, maintaining a high index of suspicion during assessments is crucial for achieving earlier diagnosis and better prognosis 8.

## Patients and Methods

This retrospective cohort study analyzed variables collected from the medical records of patients diagnosed with colon and rectal cancer at King Khalid University Hospital (KKUH). The research was conducted at KKUH, a multidisciplinary hospital that provides primary, secondary, and tertiary care. Patients diagnosed between January 1, 2016, and December 31, 2020—the first five full years of the hospital's electronic healthcare records system—were included. We identified the study cohort using the tumour database at the Research Unit of the Oncology Center at the hospital. Of the 698 entries received, 43 were excluded due to insufficient information in medical records, mislabeling in the cancer registry, or substantial therapy and care having been received elsewhere. After applying these exclusion criteria, 655 patients were included in the analysis.

A secure digital form was used to consolidate information gathered from patient files, including demographics, smoking history, family history of cancer, and symptoms. For each symptom, three options were provided: yes, no, or information not available.

This study was approved by the Institutional Review Board, Health Sciences Colleges Research on Human Subjects, King Saud University, College of Medicine, with approval numbers 20/0815/IRB on November 10, 2020, and 22/0919/IRB on November 13, 2022. Patients' consents to use data for research was obtained from patients or legal guardians (if applicable) to publish information as appropriate as per the policies and procedures of the institution and local regulations. The study was conducted in accordance with the ethical guidelines of the Declaration of Helsinki.

The data were analyzed using the R programming language for statistical computing 9. The following packages were used: tidyverse 10, survival 11,12, ggfortify 13,14, and survminer 15.

## Results

**Figure 1:**
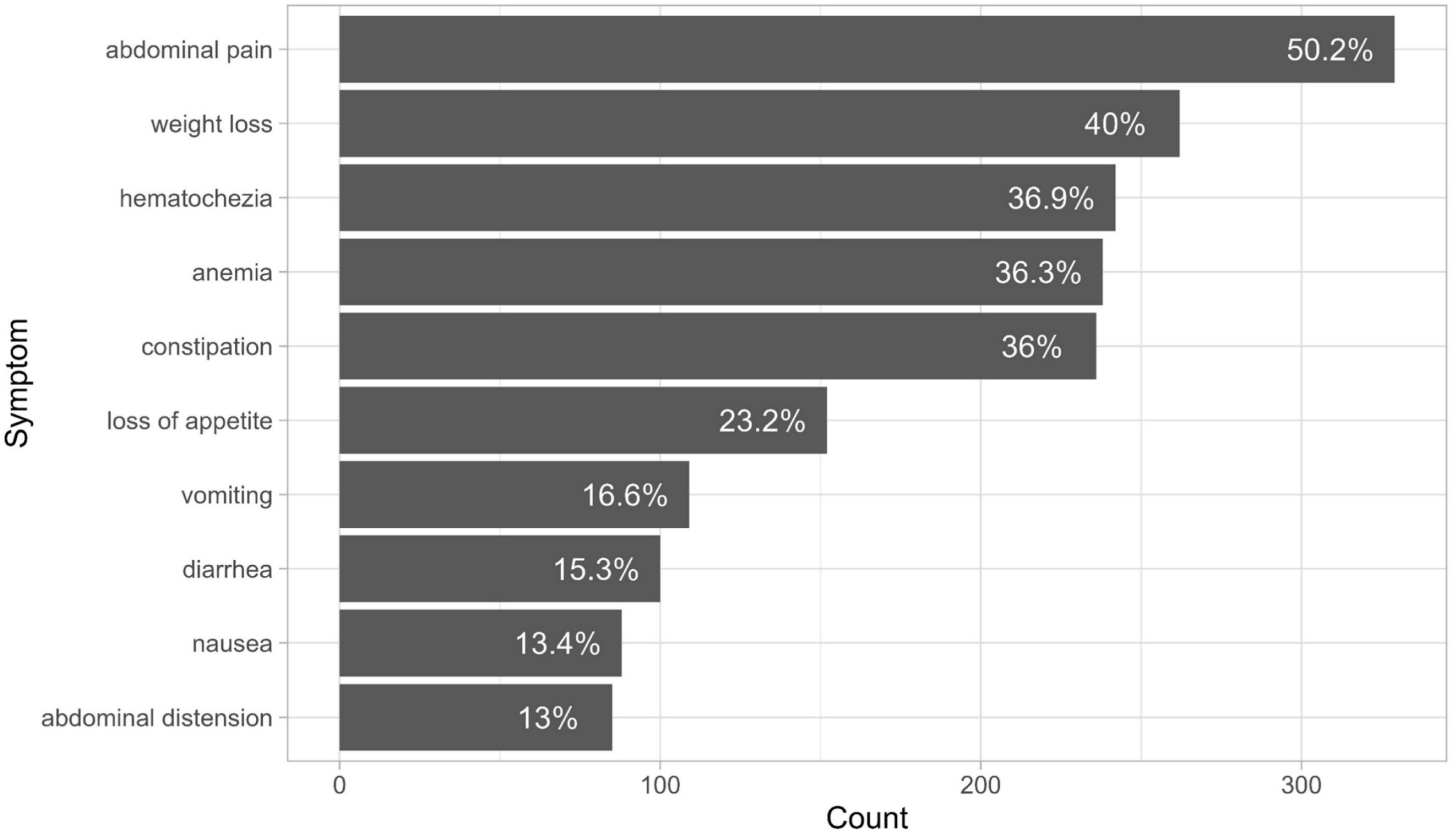
Ten most common presenting symptoms, sorted from most common to least common.

Data on 655 patients were collected for the analysis in this paper. The median age at diagnosis was 58 years (mean = 57.9, SD = 13.8). Of the 655 patients, 144 (22%) were younger than 50 years. The cohort consisted of 371 males (56.6%) and 284 females (43.4%). In terms of smoking status, 47 (7.2%) were smokers, 36 (5.5%) were ex-smokers, and 150 (22.9%) were non-smokers, smoking status for the rest of the patients was unclear. 39.4% of the patients were diabetic, 38.6% were hypertensive, and 17.7% were diagnosed with dyslipidaemia. The most common presenting symptom was abdominal pain, followed by weight loss, hematochezia (rectal bleeding or blood in stool), and anemia, which is defined a hemoglobin level of below 12 g/dl for females and 13 g/dl for males according to World Health Organization guidelines 16. Figure 1 and Table 1 show the ten most common presenting symptoms, sorted from most to least common.

Scoping was the most common method of diagnosis, used for 450 patients (68.7%), followed by surgery for 49 patients (7.5%). Additionally, 50 patients (7.6%) had a history of CRC in a first-degree family member, while 33 patients (5%) had a history of non-CRC in a first-degree family member.

A total of 118 patients (18%) had documented initial presentation to the ER, and only 22 patients (3.4%) were documented as presenting at a family medicine clinic. The median duration of symptoms for all patients was 90 days; however, patients presenting through the ER had a longer median duration of symptoms, at 120 days. Only 19 patients (2.9%) had a documented history of previous malignancy, while 55% were documented as having no previous history of malignancy.

Patients' hemoglobin levels at presentation and diagnosis were reviewed. At diagnosis, the median hemoglobin level was 10.9 g/dL, and the mean was 11.1 g/dl. The median values of Carcinoembryonic antigen (CEA) at diagnosis was 5.84 µg/. Table 2 shows the stage distribution according to the eighth edition AJCC cancer staging.

Approximately half of the patients had no metastasis at diagnosis (304, 46.1%). The most common site for metastasis was the liver (124, 18.9%), followed by the peritoneum (58, 8.9%). Tumor grading is detailed in Table 3.

**Figure 2:**
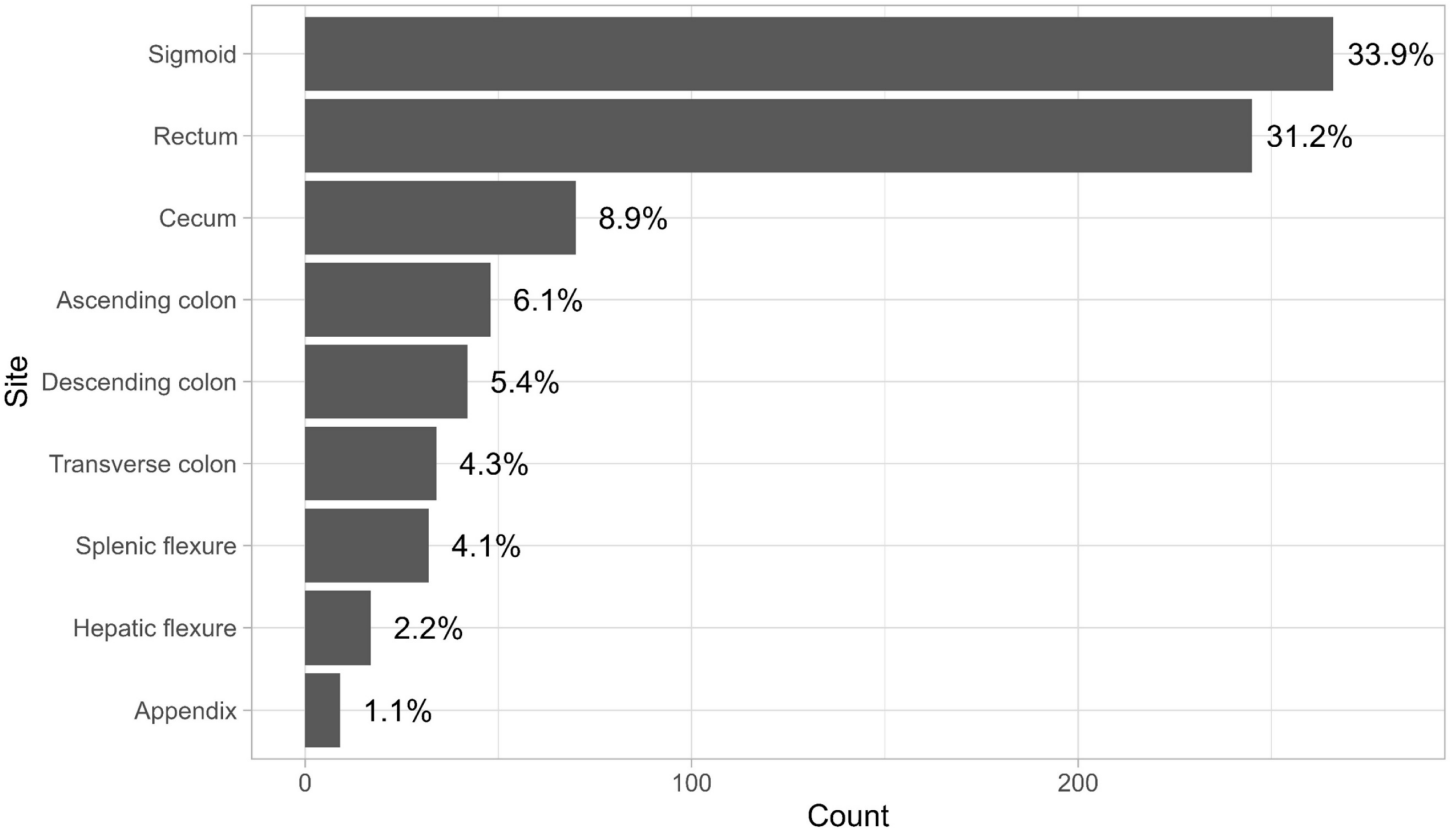
Anatomical sites of disease.

The anatomical sites of tumor involvement showed a wide range, with the transverse colon and sigmoid colon being the most common sites. Figure 2 illustrates that the most prevalent combination observed was between the rectum and sigmoid colon, followed by the combination of the descending colon and sigmoid colon, and then the ascending colon and cecum. 88% of the patients who underwent surgery underwent it electively and not as an emergency procedure. 4.8% of the patients were documented to have developed surgical wound site infection after their surgery, and about 4.25% developed either an abdominal or pelvic abscess. Only 1.5% developed an anastomotic leak.

**Figure 3:**
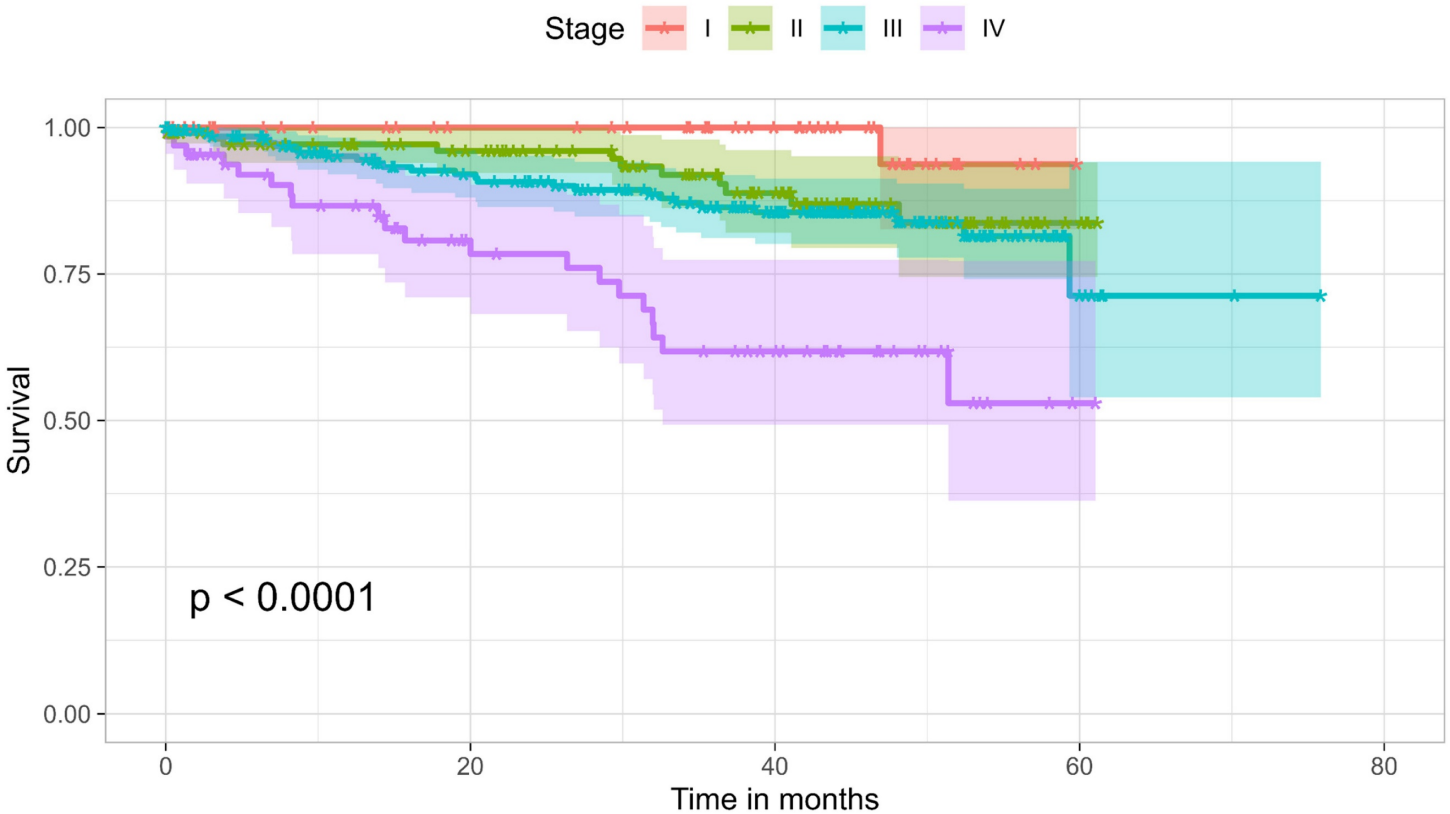
Kaplan-Meier survival curves of the cohort, comparing the four stages.

At the time of data collection, 136 patients (20.8%) had recorded death in their health records. The overall survival rate over three years was 77.6%. Survival by stage was as follows: 100% for Stage I, 91.9% for Stage II, 86.4% for Stage III, and 61.8% for Stage IV, with a significant difference (p < 0.0001). Figure 3 shows a Kaplan-Meier analysis of the cohort comparing the four different stages. The analysis showed no significant difference in survival between age groups of younger than 50 years and 50 years and older, nor with gender, smoking history, or site of disease. However, its seems that the higher the number of symptoms, the worse the outcome (p= 0.0076). This may be a surrogate for stage, but may also be an independent predictor of outcome. The absence of difference between the different age groups could be attributed to the baseline condition of the patient population, with nearly 78% either diabetic or hypertensive.

## Discussion

Data on 655 CRC patients in the KKUH cancer registry were analyzed. The most common presenting symptoms were abdominal pain (50.2%), weight loss (40%), and hematochezia (36.9%). The findings of this study are consistent with a similar study that reported abdominal pain (68%), rectal bleeding (62%), and weight loss (55%) as the three most common symptoms 17. The median age at diagnosis was 58 years, with 22% of patients being under 50 years of age at the time of diagnosis. These findings align with other local studies, suggesting that CRC in Saudi Arabia may present at an earlier age compared to other countries 17, 18.

Only one patient in the cohort was diagnosed through a screening colonoscopy, and three were diagnosed incidentally. Only 2.1% of patients presented through a primary care clinic, highlighting a gap in CRC screening access in Saudi Arabia. The role of primary care in CRC detection is crucial and directly impacts patient prognosis. A previous study found that patients with red flag symptoms referred from primary care clinics after three months had a significantly worse prognosis than those referred within two weeks, emphasizing the need for timely referrals 19. One of the more striking findings from this study was that only one patient was referred from a screening clinic. This shows a significantly low uptake of screening for colorectal cancer. Factors as waiting time, inability to access timely screening, and lack of knowledge about different screening methods all contribute to this. A city-wide or nationwide structured screening program can significantly increase the uptake, with protected slots for endoscopy or CT scans for screening eligible cases only. Educating patients about other options including fecal immunochemical test (FIT), fecal occult blood test (FOBT), or computed tomography colonography, is also equally important and valuable, and any added cost incurred from such programs is effectively paid for by long-term earnings from early detection and higher remission and survival rates.

The overall survival rate over three years was 77.6%, with survival rates at stages I, II, III, and IV being 100%, 91.9%, 86.4%, and 61.8%, respectively. This is higher than a similar local retrospective study on patients diagnosed and managed between 2009 and 2017, which reported an overall three-year survival rate of 65% 20.

This study has several limitations. First, the sample population is drawn from only one hospital, KHUH, which may limit the generalizability of the findings. Second, data were collected from patient electronic records, which can vary in accuracy and completeness. Additionally, a sizable portion of the included patients were referred from other hospitals, often with incomplete documentation, further limiting the quantity of data available for analysis. Third, since some patients were diagnosed less than five years ago, the five-year survival rate, a standard outcome measure for CRC, could not be accessed. Thus, long-term survival outcomes could not be fully evaluated, which may affect the prognostic implications of the study's findings. Future studies could address these limitations by including a more diverse sample from various regions and hospitals in Saudi Arabia and using a standardized, validated data collection tool.

## Figures and Tables

**Table 1:** Presenting symptoms, sorted from most common to least common

Symptom	Yes	No	Information not available
Abdominal pain	329 (50.2%)	76 (11.6%)	250 (38.2%)
Weight loss	262 (40%)	121 (18.5%)	272 (41.5%)
Hematochezia	242 (36.9%)	138 (21.1%)	275 (42%)
Anemia by lab	238 (36.3%)	111 (16.9%)	306 (46.7%)
Constipation	236 (36%)	96 (14.7%)	323 (49.3%)
Loss of appetite	152 (23.2%)	100 (15.3%)	403 (61.5%)
Vomiting	109 (16.6%)	192 (29.3%)	354 (54%)
Diarrhea	100 (15.3%)	114 (17.4%)	441 (67.3%)
Nausea	88 (13.4%)	128 (19.5%)	439 (67%)
Abdominal distension	85 (13%)	44 (6.7%)	526 (80.3%)
Bowel obstruction	71 (10.8%)	51 (7.8%)	533 (81.4%)
Lethargy or fatigability	61 (9.3%)	12 (1.8%)	582 (88.9%)
Melena	60 (9.2%)	102 (15.6%)	493 (75.3%)
Fever	43 (6.6%)	187 (28.5%)	425 (64.9%)
Night sweats	25 (3.8%)	80 (12.2%)	550 (84%)
Tenesmus	25 (3.8%)	8 (1.2%)	622 (95%)
Perianal pain	21 (3.2%)	8 (1.2%)	626 (95.6%)
Back pain	15 (2.3%)	5 (0.8%)	635 (96.9%)
Bowel perforation	13 (2%)	45 (6.9%)	597 (91.1%)
Weakness	10 (1.5%)	8 (1.2%)	637 (97.3%)
Metastasis symptoms	5 (0.8%)	3 (0.5%)	647 (98.8%)
Asymptomatic incidental	3 (0.5%)	545 (83.2%)	107 (16.3%)
Anal pruritus or discharge	2 (0.3%)	5 (0.8%)	648 (98.9%)
Asymptomatic screening	1 (0.2%)	545 (83.2%)	109 (16.6%)

**Table 2:** Stage distribution according to the eighth edition of the AJCC cancer staging for those who had enough data for staging (n= 500, 76.4%)

Stage	Count n (%) *
Stage I	*56 (8.6%)*
Stage II	*127 (19.4%)*
Stage III	*237 (36.2%)*
Stage IV	*80 (12.2%)*

**Table 3:** Tumor grading

Tumor grade	Count (%)
Information not found	89 (13.6%)
Undifferentiated to poorly differentiated	1 (0.15%)
Poorly differentiated	31 (4.7%)
Poorly to moderately differentiated	20 (3%)
Moderately differentiated	473 (72.2%)
Moderately to well-differentiated	21 (3.2%)
Well-differentiated	20 (3%)
